# Supplementary material for: Can neural networks benefit from objectives that encourage iterative convergent computations? A case study of ResNets and object classification
Source: PLoS One. 2024 Mar 21;19(3):e0293440. doi: 10.1371/journal.pone.0293440 (PMC10956829; doi:10.1371/journal.pone.0293440)
Supplement: S1 Appendix — It also details a few variations on gradient coupling that were explored. As detailed in the appendix, the findings on these variations were consistent with the results presented in the main article. (PDF) [file pone.0293440.s001.pdf]

Supplementary material

S1 Network training

S1.1 Software

We trained the networks using PyTorch (1), PytorchLightning, numpy (2), and pandas in Python (3). The remaining analysis was conducted in R (4), using ggplot2 (5), dplyr (6), tidyr (7), patchwork (8), and DescTools (9). The implementation of ResNet-104 was significantly supported by an existing implementation (10).

S1.2 Datasets

We trained and evaluated the networks on four datasets: CIFAR-10, CIFAR-100 (11), MNIST (12), and Digitclutter (13). All images were normalized before being provided to the network. For CIFAR-10, we used a training/validation sample split of 45000/5000, augmented the data by random cropping and random horizontal flips during training. For MNIST, we used a split of 50000/10000, and augmented the data by random cropping. Finally, for Digitclutter, we used between two and five overlapping digits (referred to as Digitclutter-3) with a split of 100000/10000 without data augmentation.

S1.3 Architecture details

As our base architecture we use a ResNet with the preactivation unit recommended by (14). This means that a residual block consists of

$$\mathrm{BN}_1 \to \mathrm{ReLU} \to \mathrm{Conv}_1 \to \mathrm{BN}_2 \to \mathrm{ReLU} \to \mathrm{Conv}_2,$$

where BN stands for batch normalization (15), ReLU for rectified linear unit, and Conv for a convolutional layer.

For CIFAR-10, CIFAR-100, and Digitclutter, this ResNet contained three stages with 16 blocks each. This is a common depth for a ResNet. For example (16) use a ResNet-110. Two stages were connected by a downsampling block, which halved width and height of the representation and doubled the number of channels. The downsampling block consisted of a residual block where the first convolutional layer had stride 2 and doubled the number of channels. The shortcut connection (otherwise simply the identity function) decreased the image resolution by taking into account only every second column and row. To increase the number of channels, the shortcut connection added a number of layers initialized as zero. This downsampling block was never removed as part of the perturbations defining the indices of iterative convergence.

This means that for a standard ResNet with 16 channels in the first stage, the second stage consisted of (16x16)-representations with 32 channels, and the third stage consisted of (8x8)-representations with 64 channels. In contrast, we only used one stage with 16 blocks for MNIST.

S1.4 Training details

We initialized our networks using Kaiming initialization (17) for the convolutional and linear weights. In the case of a recurrent initialization, these were equal across residual

blocks of a stage. In the case of a non-recurrent initialization, they were drawn independently for each block. We initialized the batch normalization's scale γ with 1 and its bias with 0.

We trained the original networks for 400 epochs using gradient descent with momentum 0.9. For CIFAR-10, we used an initial learning rate of 0.1, which was divided by 10 after 200 and 300 epochs. For CIFAR-100 and Digitclutter, we trained the networks for 200 epochs and used an initial learning rate of 0.1, which was divided by 10 after 100 and 150 epochs. For MNIST, we used an initial learning rate of 0.025, which was divided by 2.5 after 200 and 300 epochs. For Digitclutter, we used an initial learning rate of 0.05, which was divided by 5 after 200 and 300 epochs. We then identified the epoch at which the network had obtained the best validation classification error and used the corresponding model for all analyses.

S2 Training variations

On CIFAR-10, we explored several variations of the training algorithm, which were consistent with the findings presented above.

S2.1 Recurrent batch normalization

(18) proposed to initialize the scale of the batch normalization γ as 0.1 in recurrent neural networks. When applying this method to softly gradient-coupled networks, we find that the method strongly improves performance for $\lambda = 0.9$, but not for any other coupling parameters (see Figure S3a).

S2.2 Triangular gradient coupling

We may generalize soft gradient coupling to the coupling rule

$$\tilde{\Delta}_t = \sum_{s=1}^{T} \kappa(s, t; \lambda) \partial_t L, \tag{1}$$

where κ is some kernel depending on the coupling parameter λ. Ordinary soft gradient coupling can be recovered with a uniform kernel

$$\kappa(s, t; \lambda) := \begin{cases} (1-\lambda) + \lambda/T & \text{if } s = t, \\ \lambda/T & \text{if } s \neq t. \end{cases} \tag{2}$$

Alternatively, we may want to couple adjacent blocks more strongly than blocks that are far away from each other. This can, for example, be achieved with a triangular kernel

$$\kappa(s, t; \lambda) := \begin{cases} (1 - 2 \cdot (1-\lambda)/T \cdot |s-t|)^+ & \text{if } \lambda \geq 0.5, \\ (1 - 1/(2 \cdot \lambda \cdot T) \cdot |s-t|)^+ & \text{if } \lambda < 0.5, \end{cases} \tag{3}$$

where

$$(\cdot)^+ = \max(\cdot, 0).$$

Again, $\lambda = 0$ corresponds to an ordinary ResNet and $\lambda = 1$ corresponds to a fully recurrent ResNet, whereas intermediate values for λ smoothly interpolate between the two.

Figure S3b shows the effect of triangular as compared to uniform gradient coupling. For a given coupling parameter, this method appears to outperform the uniformly gradient-coupled network, but neither method outperforms an ordinary ResNet.

S2.3 Start coupling at later layers

Finally, we uncoupled the first five residual blocks and only constrained the remaining blocks. For the fully recurrent case, this corresponds to the implementation by (19). We also studied the effect of this late coupling together with a batchnorm initialization of $\gamma = 0.1$. In both cases, the late coupling means that the coupled networks are better than coupled networks which start coupling at block 0, but still worse than an ordinary ResNet.

S2.4 Iterative convergence indices

Figure S4 demonstrates that for the extended training variations, higher coupling parameters generally tend to increase the Convergence Index and decrease the Divergence Index, as well. The extended training variations also predominantly yield networks with a Recurrence Index around 1.

References

1. Paszke A, Gross S, Massa F, Lerer A, Bradbury J, Chanan G, et al. PyTorch: An Imperative Style, High-Performance Deep Learning Library. In: Wallach H, Larochelle H, Beygelzimer A, Alché-Buc Fd, Fox E, Garnett R, editors. Advances in Neural Information Processing Systems 32. Curran Associates, Inc.; 2019. p. 8024–8035. Available from: `http://papers.neurips.cc/paper/9015-pytorch-an-imperative-style-high-performance-deep-learning-library.pdf`.

2. Oliphant TE. A guide to NumPy. vol. 1. Trelgol Publishing USA; 2006.

3. van Rossum G, Drake FL. Python 3 Reference Manual. Scotts Valley, CA: CreateSpace; 2009.

4. R Core Team. R: A Language and Environment for Statistical Computing. Vienna, Austria: R Foundation for Statistical Computing; 2019. Available from: `https://www.R-project.org/`.

5. Wickham H. ggplot2: Elegant Graphics for Data Analysis. Springer-Verlag New York; 2016. Available from: `https://ggplot2.tidyverse.org`.

6. Wickham H, François R, Henry L, Müller K. dplyr: A Grammar of Data Manipulation; 2019. Available from: `https://CRAN.R-project.org/package=dplyr`.

7. Wickham H, Henry L. tidyr: Tidy Messy Data; 2019. Available from: `https://CRAN.R-project.org/package=tidyr`.

8. Pedersen TL. patchwork: The Composer of Plots; 2019. Available from: `https://CRAN.R-project.org/package=patchwork`.

9. Signorell A. DescTools: Tools for Descriptive Statistics; 2020. Available from: `https://cran.r-project.org/package=DescTools`.

10. Idelbayev Y. Proper ResNet Implementation for CIFAR10/CIFAR100 in PyTorch; 2018. Available from:
`https://github.com/akamaster/pytorch_resnet_cifar10`.

11. Krizhevsky A. Learning Multiple Layers of Features from Tiny Images; 2009.

12. LeCun Y, Cortes C, Burges CJ. MNIST handwritten digit database. 2010;.

13. Spoerer CJ, McClure P, Kriegeskorte N. Recurrent Convolutional Neural Networks: A Better Model of Biological Object Recognition. Frontiers in Psychology. 2017;8.

14. He K, Zhang X, Ren S, Sun J. Identity mappings in deep residual networks. In: European conference on computer vision. Springer; 2016. p. 630–645.

15. Ioffe S, Szegedy C. Batch Normalization: Accelerating Deep Network Training by Reducing Internal Covariate Shift. In: International Conference on Machine Learning; 2015. p. 448–456.

16. He K, Zhang X, Ren S, Sun J. Deep residual learning for image recognition. In: Proceedings of the IEEE conference on computer vision and pattern recognition; 2016. p. 770–778.

17. He K, Zhang X, Ren S, Sun J. Delving deep into rectifiers: Surpassing human-level performance on imagenet classification. In: Proceedings of the IEEE international conference on computer vision; 2015. p. 1026–1034.

18. Cooijmans T, Ballas N, Laurent C, Gülçehre [U+FFFD] Courville A. Recurrent batch normalization. In: International Conference on Learning Representations; 2017.

19. Jastrzebski S, Arpit D, Ballas N, Verma V, Che T, Bengio Y. Residual Connections Encourage Iterative Inference. In: International Conference on Learning Representations; 2018.
