# Supplementary figures and images for: Can neural networks benefit from objectives that encourage iterative convergent computations? A case study of ResNets and object classification
Source: PLoS One. 2024 Mar 21;19(3):e0293440. doi: 10.1371/journal.pone.0293440 (PMC10956829; doi:10.1371/journal.pone.0293440)

a

# channels 8 16 32 64

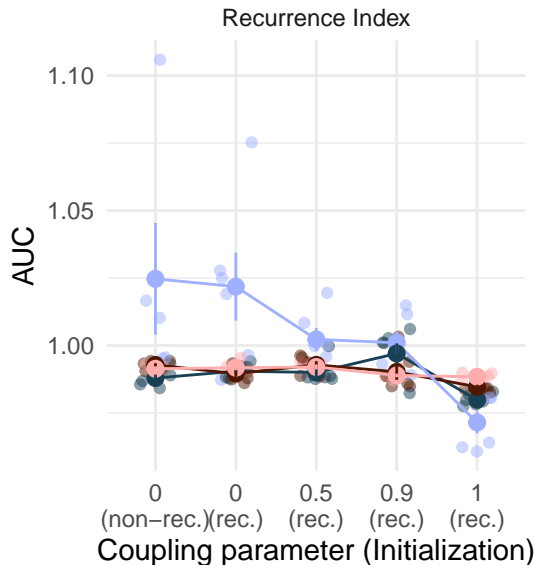

b

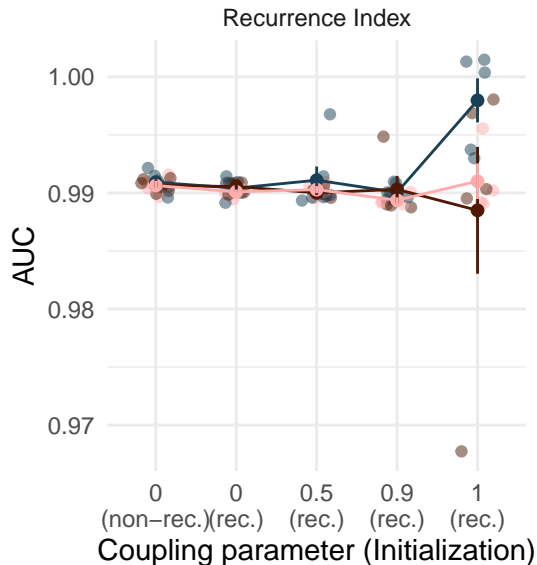

Supplement: S1 Fig — a Recurrence Index for gradient-coupled ResNets. b Recurrence Index for improperly convergent ResNets. (PDF) [file pone.0293440.s002.pdf]

AUC

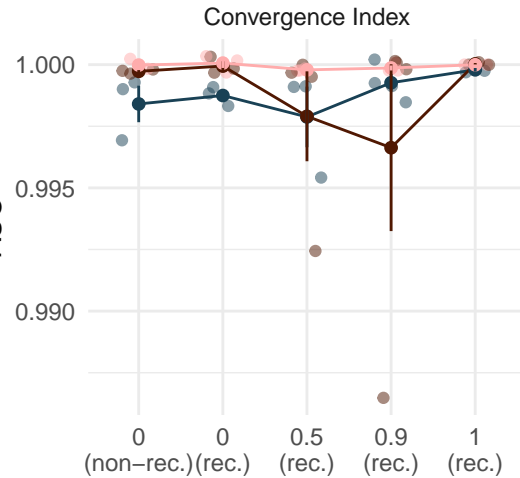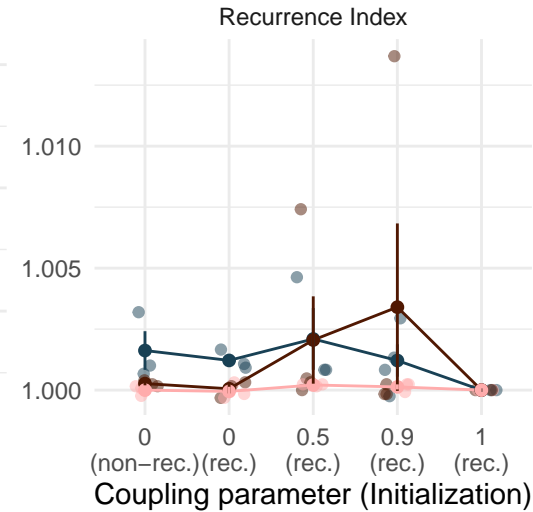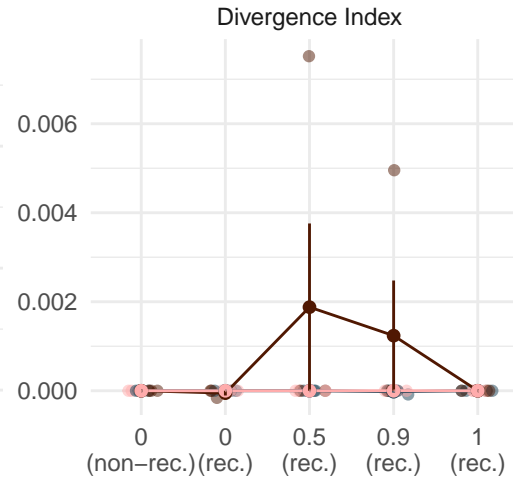

Error rate on CIFAR-10

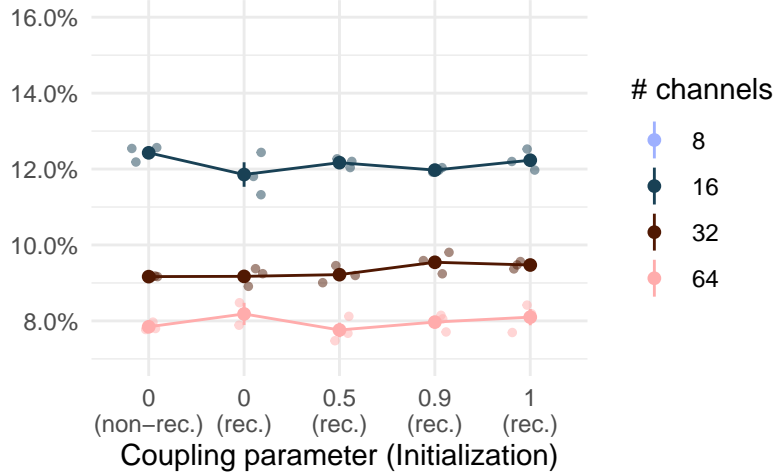

Supplement: S2 Fig — a The indices of iterative convergence demonstrate that the PCRs indeed converge. b As the error rate on CIFAR-10 indicates, PCRs tend to perform a bit worse than the improperly convergent ResNets we studied in the main article. (PDF) [file pone.0293440.s003.pdf]

a

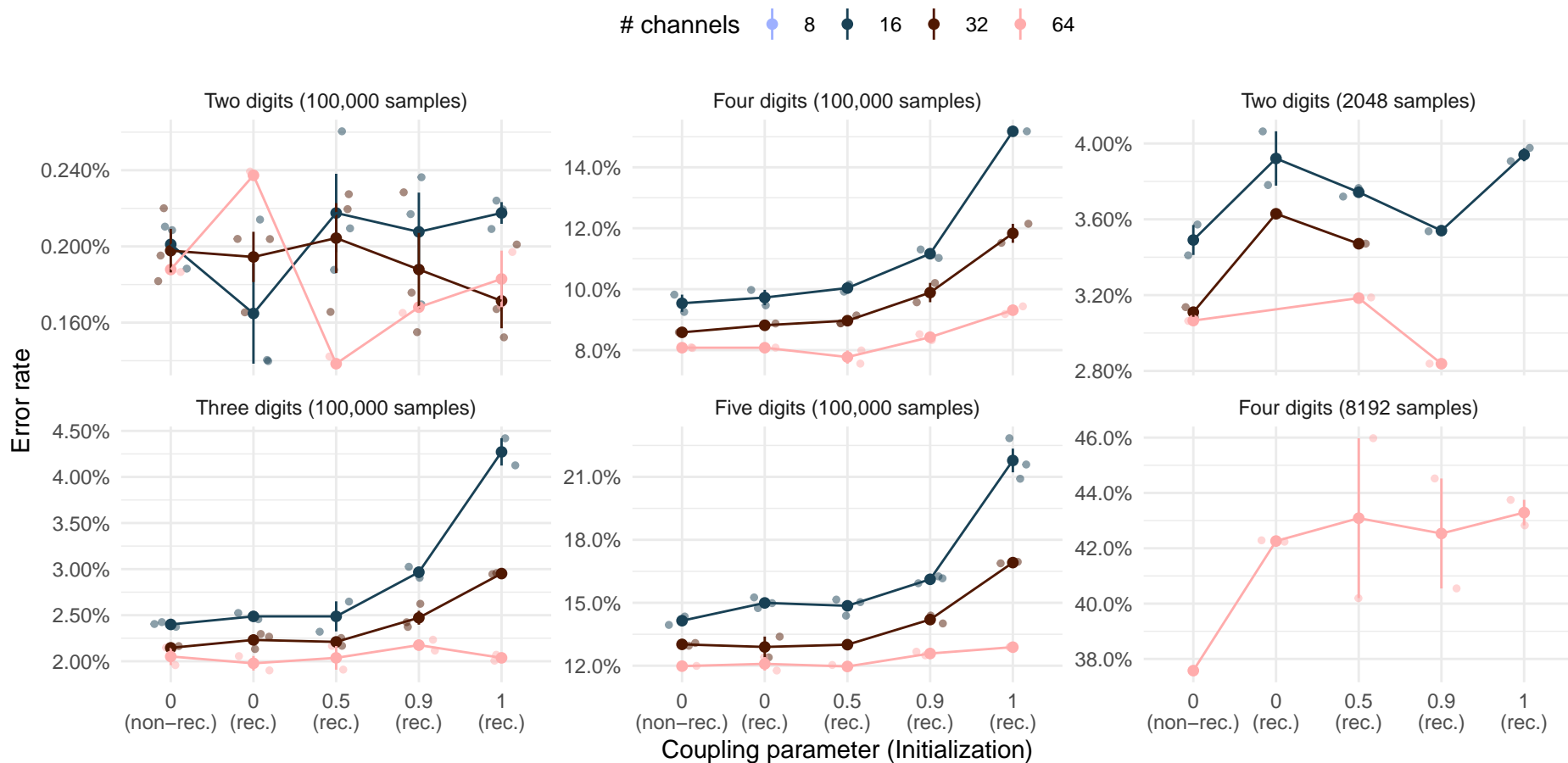

b

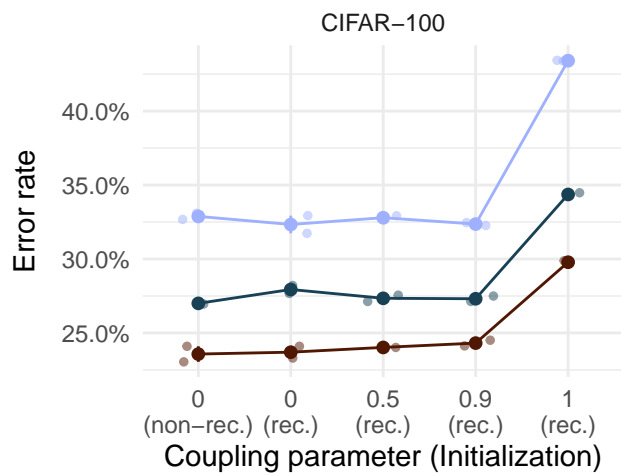

c

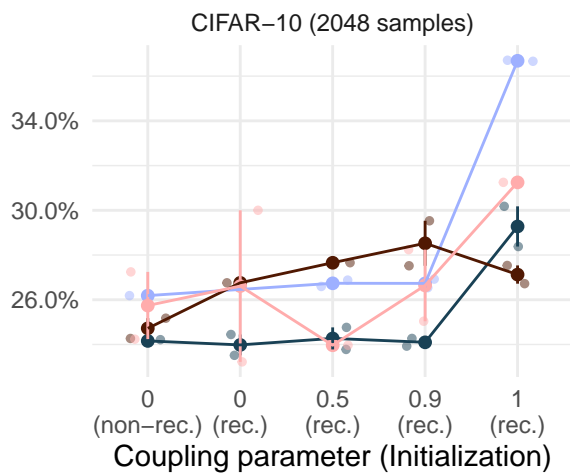

d

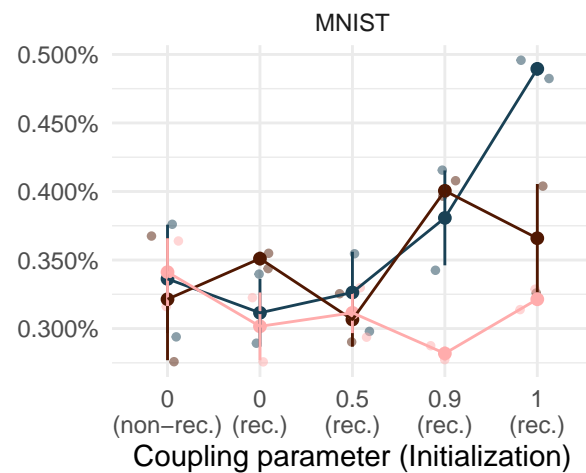

Supplement: S3 Fig — a Performance of gradient-coupled ResNets on variations of Digitclutter with a different number of overlapping digits and different size of training data. b Performance of gradient-coupled ResNets on CIFAR-100, c CIFAR-10 with few training data, and d MNIST. (PDF) [file pone.0293440.s004.pdf]

a

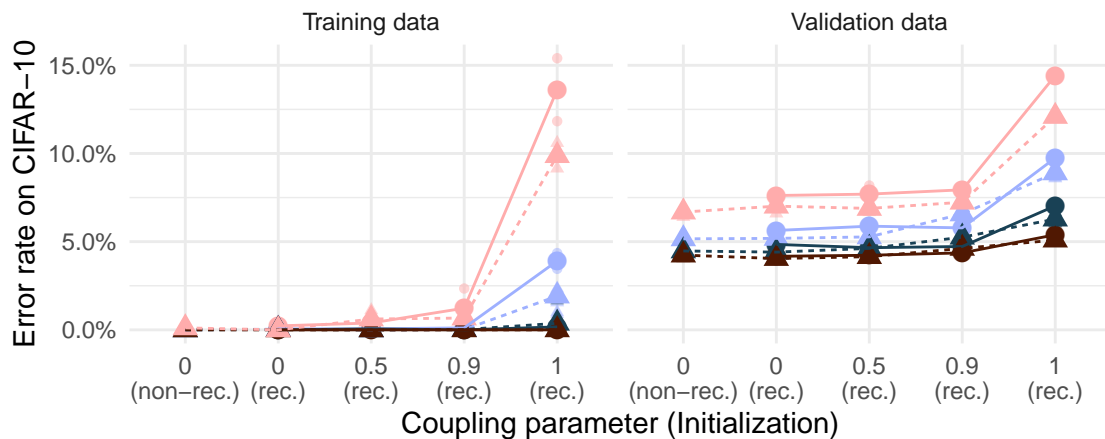

b

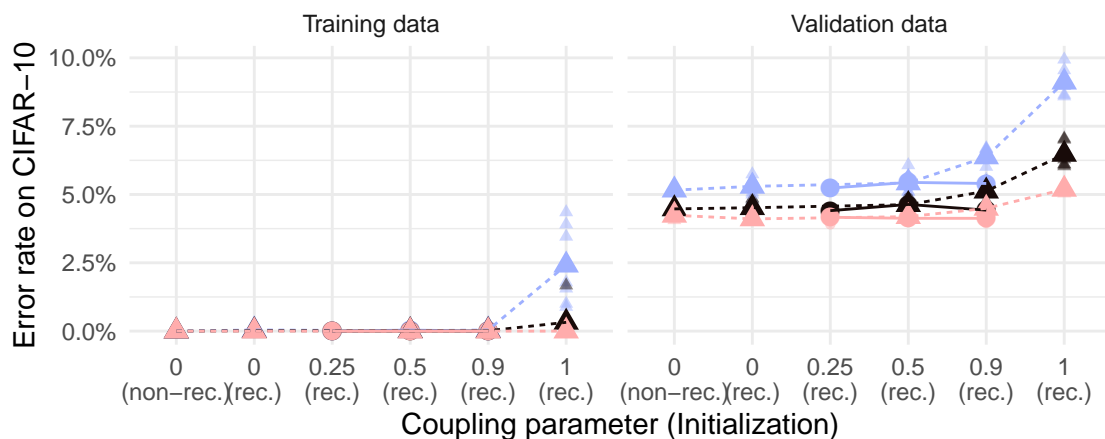

c

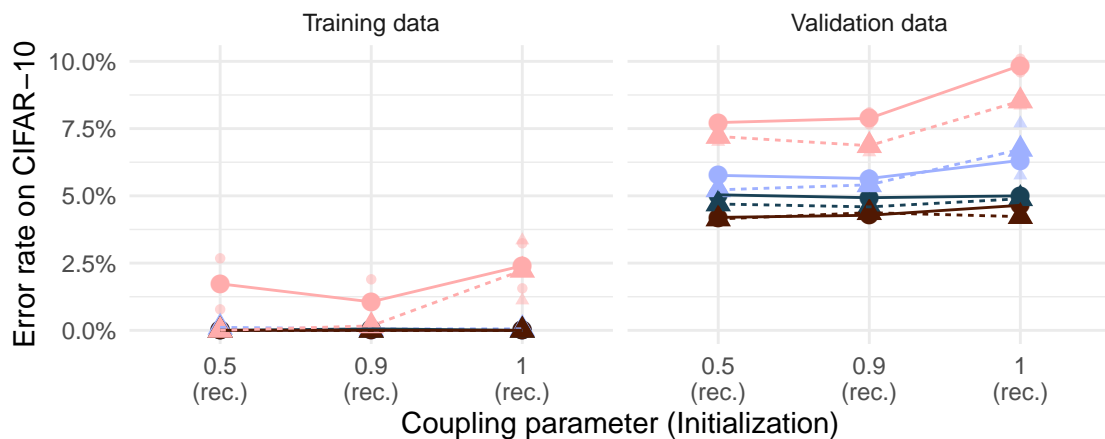

Supplement: S4 Fig — a The effect of initializing batchnorm with γ = 0.1 instead of γ = 1. b The effect of using a triangular kernel for gradient coupling instead of a uniform kernel. c A variation of gradient coupling where the first five blocks in each stage were uncoupled. (PDF) [file pone.0293440.s005.pdf]

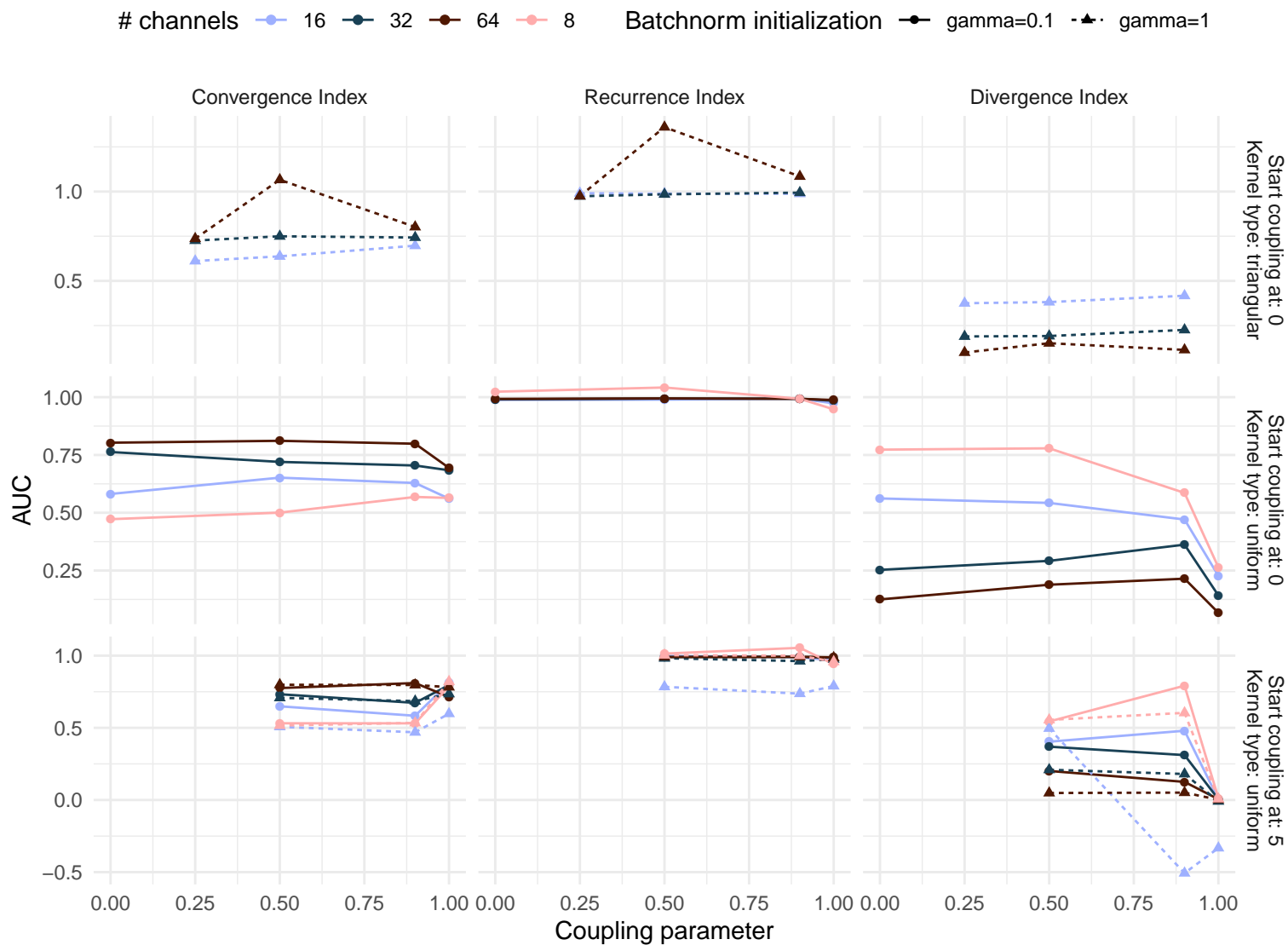

Supplement: S5 Fig — (PDF) [file pone.0293440.s006.pdf]

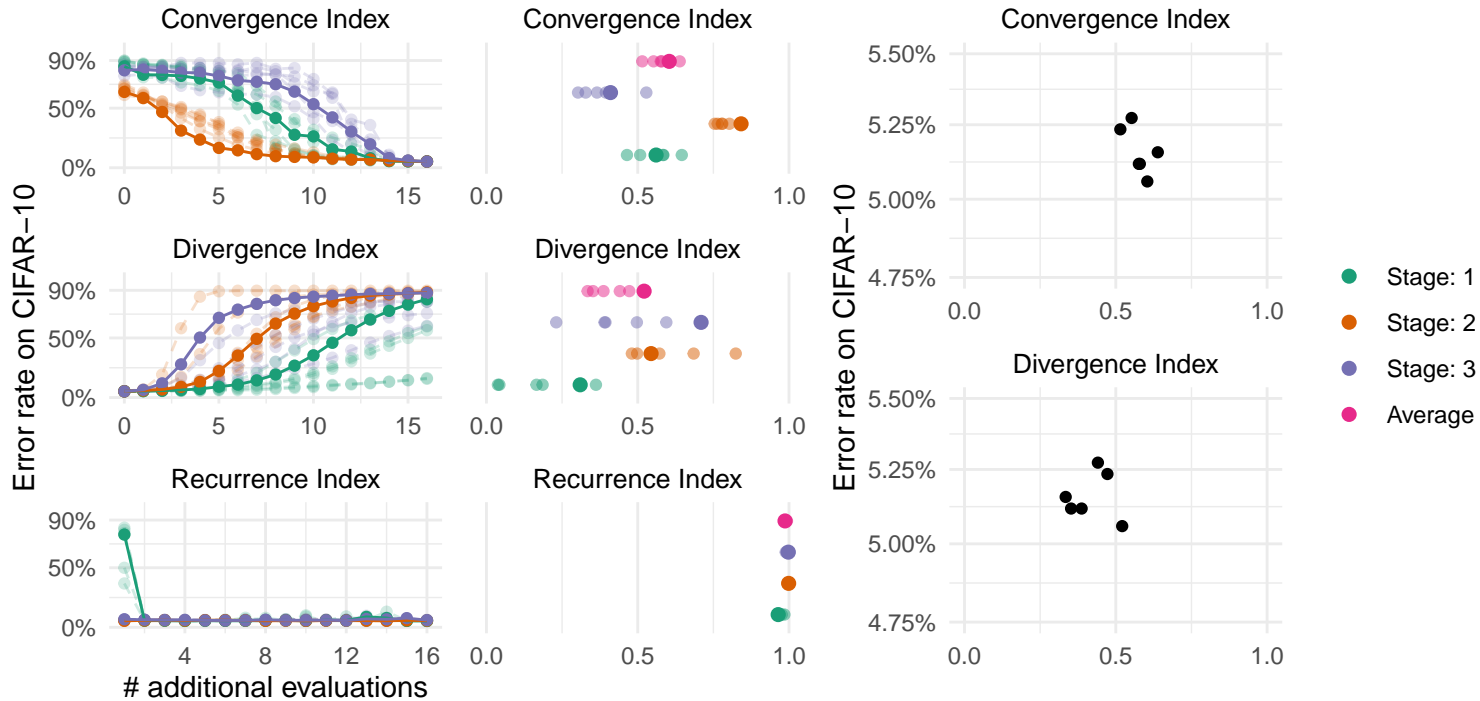

Supplement: S1 File — This repository contains the code to train the models, the resulting performance metrics, and code to analyse these metrics. (ZIP) [file pone.0293440.s007.zip › plos-one-submission/figures/fig-2-raw.pdf]

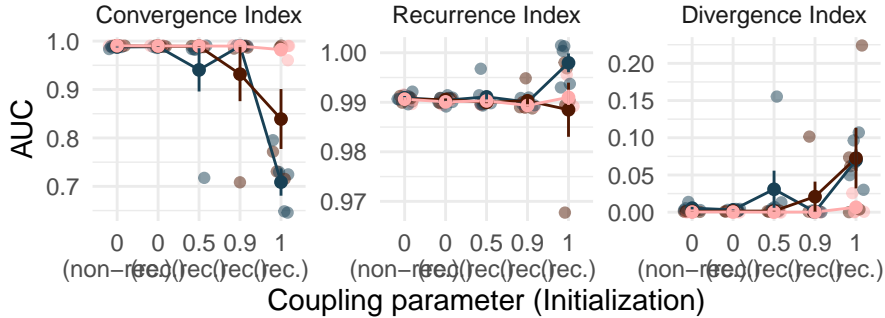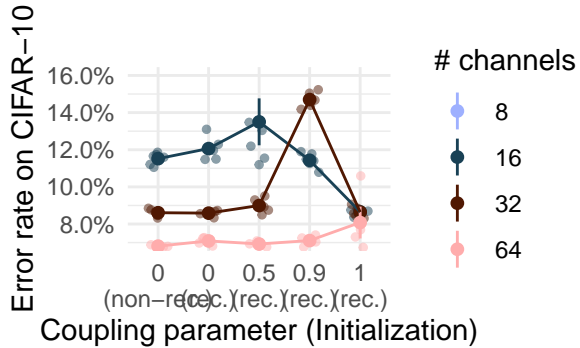

Supplement: S1 File — This repository contains the code to train the models, the resulting performance metrics, and code to analyse these metrics. (ZIP) [file pone.0293440.s007.zip › plos-one-submission/figures/fig-4-raw.pdf]

a

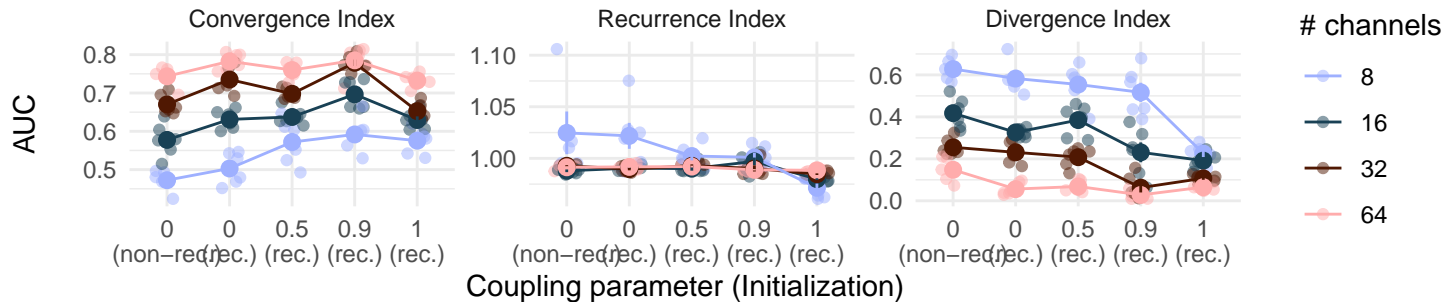

b

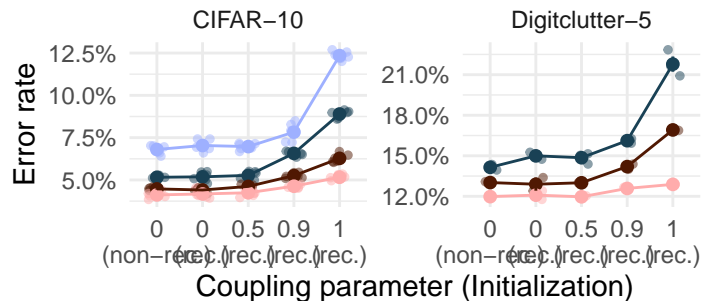

c

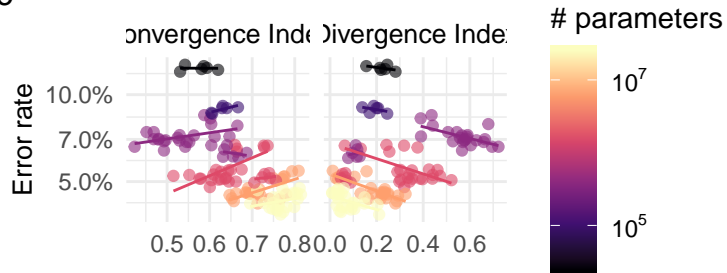

Supplement: S1 File — This repository contains the code to train the models, the resulting performance metrics, and code to analyse these metrics. (ZIP) [file pone.0293440.s007.zip › plos-one-submission/figures/fig-3-raw.pdf]
